# The Impact of Land-Based Physical Activity Interventions on Self-Reported Health and Well-Being of Indigenous Adults: A Systematic Review

**DOI:** 10.3390/ijerph18137099

**Published:** 2021-07-02

**Authors:** Fatima Ahmed, Aleksandra M. Zuk, Leonard J.S. Tsuji

**Affiliations:** 1 Department of Physical and Environmental Sciences, University of Toronto, Toronto, ON M1C 1A4, Canada; amz4@queensu.ca (A.M.Z.); leonard.tsuji@utoronto.ca (L.J.S.T.); 2 School of Nursing, Faculty of Health Sciences, Queen’s University, Kingston, ON K7L 3N6, Canada

**Keywords:** Indigenous, self-reported health, well-being, physical activity, traditional activities, adults, health

## Abstract

For many Indigenous communities, decreased participation in traditional land-based activities has led to higher rates of chronic disease and a decrease in well-being. This systematic review explores how traditional land-based activities impact self-reported health and well-being of Indigenous adults, using Indigenous and Western perspectives. A search of three electronic databases (PubMed, Scopus, and Web of Science) identified nine studies which explored the experiences and perspectives of Indigenous adults taking part in land-based subsistence and ceremonial activities. A thematic analysis of these studies identified many interconnected physical, spiritual, mental, emotional, and community benefits. Community engagement throughout all stages of the interventions was an important factor in effectively addressing challenges and barriers stemming from colonization, decreased knowledge transfer, and increased use of technology. Participants reported developing more effective stress management techniques, a greater awareness of modifiable risk factors along with increased engagement with Elders. Ultimately, land-based subsistence and ceremonial activities were identified as playing an influential role in the lives of Indigenous adults. The involvement of community members allowed for the development of more culturally relevant interventions. Future community-specific research is needed to increase engagement in traditional physical-activities, improve well-being and overall reduce the risk of chronic disease.

## 1. Introduction

Connection with the land has always been a fundamental part of the health and well-being of Indigenous communities [1]. The land not only provides a source of sustenance, but is a crucial part of maintaining cultural identity, along with benefits at the individual, familial and community level [1,2]. The notion of cultural identity relating to well-being was first articulated in research by Durkheim [3], who stressed the importance of culture in defining an individual. This idea, however, has been longstanding in Indigenous teachings such as the medicine wheel, which is used in some cultures, and serves as a metaphor for a diverse range of spiritual concepts. One of those concepts is the interconnectedness of internal and external factors which must align to achieve overall well-being [4,5]. In Western approaches to health, it has only been recently acknowledged that Indigenous culture, identity, and lifestyle are deeply rooted in their land, and ultimately their well-being [6,7]. This was observed in a study by Hossain and Lamb [5], which found that providing support and opportunities for traditional activities, such as arts, crafts, hunting and fishing, led to greater psychological well-being for Indigenous people in Canada living in non-metropolitan areas.

Several studies have suggested that being disconnected from traditional practices has led to poorer physical and mental health, and overall lower levels of well-being [2,5,8]; colonization has led to this disconnect. Colonization is defined as the settlement and establishment of control over Indigenous people and their lands [8,9]. Settler colonialism, a mode of colonization, encompasses the systemic removal of Indigenous people from their ancestral lands and the disruption of the transmission of Indigenous culture and knowledge in countries such as Canada, the United States and Australia [9]. The impacts of settler colonialism have led to decreased participation in land-based activities [9,10], ultimately leading to high rates of health disparities amongst Indigenous populations compared to those of non-Indigenous populations [8,10,11,12,13,14]. The prevalence of these health disparities is in part a result of decreased physical activity, poor quality of diet and overall stress [8,11,12,13,15,16,17,18,19,20,21,22,23]. In the United States of America (USA), cardiovascular diseases such as coronary artery disease have some of the highest mortality rates amongst Native Hawaiians [13,24,25]. Similarly, in the United Houma Nation (UHN), 53% experience cardiovascular disease, vs. 5.8% of the US white population [9]. Among the Choctaw Nation, obesity rates, a risk factor for diabetes, are at 53% versus 39% for the average population of the USA [26]. Type 2 diabetes mellitus in Indigenous populations relative to their corresponding non-Indigenous communities, have been observed to be two to four times higher within the Navajo in the USA [11] and three to five times higher within First Nations in Canada [27]. Understanding factors leading to these rates through individual perceptions and experiences is crucial when addressing disparities of health and well-being amongst communities [14]. Studies have shown other factors such as climate change [4], industry development [20] and increased use of technology [28] have also caused significant changes to the lifestyle and diets of Indigenous populations globally [11,15,29,30]. These factors have caused changes to the land and disrupted traditional activities such as hunting, trapping and fishing [6,29,30]. These changes have also contributed to poorer diets within communities, causing a reliance on more store-bought foods, and decreased consumption of traditional food such as fish, which is rich in essential nutrients [11]. Partaking in subsistence activities is not only seen as sustenance but also ceremonial, as they connect an individual to the land and their community [6,15]. Other forms of ceremony and spiritual practices such as hula, pow-wow, the gathering of medicine and community-specific ceremonies have been used for healing and improving the different aspects of health [13,24,25,31]. Although there has been research showing the benefits of physical activity on health and well-being [12,13,16], when addressing the health disparities amongst Indigenous populations, more evidence is needed to assess the potential impacts of traditional land-based physical activities in health interventions. Understanding how individuals learn about their culture through their experience, also known as enculturation, has also been associated with better health outcomes within Indigenous communities [2,8,14,32]. Therefore, partaking in these activities is not only crucial to sustaining life, but is also treatment and healing from a more holistic perspective. 

From an Indigenous perspective, sustaining health is not solely the absence of disease or a singular focus on physical health, as key within a Western approach [15] but also requires care given to spiritual, mental, physical, and emotional aspects of an individual and community [2]. Western approaches to health also view individuals in isolation, whereas Indigenous approaches consider individuals as components of the whole with obligations to give, teach, share, and care for their community [10]. The use of practices outside the conventional medical practices are referred to as traditional, complementary and integrative medicines (TCIM) [33]. Increasingly, there has been exploration of using both Western and traditional Indigenous knowledge when working with Indigenous populations [34]. Using complementary approaches to understand health and wellness outcomes [35]—recently coined as “two-eyed seeing” or *Etuaptumumk* by Mi’kmaq Elders Albert, and Murdena Marshall [36]—allows for the inclusion of the diverse perspectives and experiences of Indigenous populations. Methods such as community-based participatory research (CBPR) which engage community members through all stages of research have been increasingly employed when addressing the health disparities faced by Indigenous populations [37,38,39]. Methods such as CBPR are important as they empower communities, reinforce the importance of self-determination, and allow for the identification of culturally relevant strategies to bring about social change. CBPR is rooted in many theories, one of which being the feminist theory. This theory seeks to empower vulnerable populations, utilize knowledge grounded in experience, and recognize local expertise and insights which cannot be fully understood from the outside [40]. As Indigenous perspectives and knowledge have been understudied and underrepresented [19,41], there is an increasing need to use methods which seek to cultivate more culturally specific health care interventions. 

Overall, there is a need for more studies to identify the potential benefits of using a complementary Indigenous and Western-biomedical approach to health to address the health and well-being disparities faced by Indigenous populations. Therefore, the aim of this systematic review was to evaluate the effects of land-based activities physical activities on the self-reported health and well-being of Indigenous adults. The inclusion of qualitative data highlighting the perspectives of Indigenous populations, which are often underrepresented in health research, was crucial to understanding how these land-based activities impacted their health and well-being. It is important to conceptualize through complementary approaches and understand the factors which make traditional land-based physical activities beneficial for adaptation into future health care interventions.

## 2. Materials and Methods

The methods for this review were guided by the Preferred Reporting Items for Systematic Reviews and Meta-Analyses (PRISMA) guidelines [42]. A comprehensive search strategy, found in the Supplementary Materials (Text S1), was developed by the authors to include relevant keywords and medical subject headings (MeSH) terms. The search strategy included terms related to Indigenous populations (Tribal, Native, etc.) or an equivalent term specific to their geographic location, traditional land-based activities (hunting, dance, etc.) and terms related to self-reported health and well-being (self-perception, hypertension, etc.). Electronic databases selected for this review were based on their coverage of topics of medicine, social science, science, and public health. The three databases used for this review were Medline (PubMed), Web of Science and Scopus. Strategies used for each database, along with their associated filters and ranges, are presented in Table S1.

### 2.1. Inclusion/Exclusion Criteria

**Figure 1:**
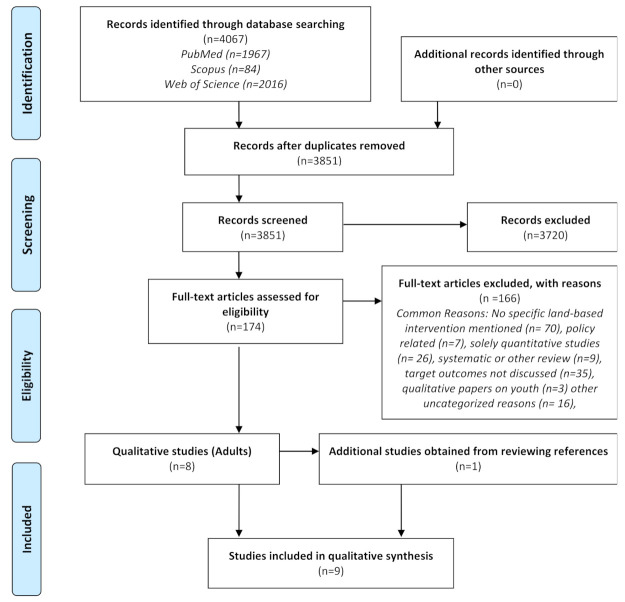
PRISMA flow diagram of search results following study selection for included publications, adapted from Moher, Liberati, Tetzlaff, Altman and The Prisma Group.

The following criteria needed to be met for studies to be included in this review. Peer-reviewed studies published online prior to 16 June 2021, were included as this was the last date searched. Studies were also limited to qualitative studies in the English language. Studies needed to explore the impacts on self-reported health of Indigenous adults participating in traditional physical activities. Results were not limited by geographic location, to allow for the inclusion of more diverse perspectives and experiences. The term Indigenous was guided by the UN identification criteria (Text S1) [43]. The review focused solely on Indigenous populations in their homelands. Adults were defined as per the United Nations [44]. The interventions included were broadly defined as traditional land-based activities to allow for inclusion of subsistence and ceremonial practices. The outcomes examined focused on self-reported health and well-being. Literature reviews, meta-analysis, and systematic reviews were excluded. Grey literature, dissertations, reports published by the government or non-government organizations and other sources that had not been subjected to the peer review process were also excluded from this review. The selection process is illustrated through the adapted PRISMA flow diagram (Figure 1) [42].

### 2.2. Study Screening and Selection 

The results from each of the three electronic databases were combined, and duplicates were removed. Qualitative studies which met the inclusion criteria, involving Indigenous populations, traditional land-based physical activities, and outcomes related to self-reported health, were selected for further appraisal. Studies were screened by their title and abstract and subsequently selected for a full text review to assess their eligibility according to the inclusion criteria. Reference lists of the included studies were reviewed to identify any relevant studies that were missed during the original search. Disagreements regarding the inclusion of studies were discussed by the authors until a consensus was achieved. 

Publications selected for the review were then appraised for the quality of the studies using the Joanna Briggs Institute (JBI) critical appraisal checklist for qualitative research [45]. This assessment helped to determine any potential biases or limitations of the papers and to enhance the quality of the review. The JBI critical appraisal checklist features 10 appraisal questions (Table S2) which cover areas of research methods, relevance, and research integrity. This tool also helps to establish the position of the authors within their publications to identify any potential biases.

### 2.3. Data Analysis

The data extraction and synthesis of the selected studies were guided by methods for the thematic synthesis of qualitative research by Thomas and Harden [46]. This method involves three stages of coding text, developing descriptive themes and subsequently analytical themes [46]. This method is effective when working with qualitative data from focus groups and interviews, to synthesize results in a transparent manner that links directly to the findings of the studies. Text from the results and findings sections of the papers, along with any participant quotes, were coded with descriptive themes focused on the outcome of self-reported health. As self-reported health is a measure that is subjective to the diverse perspectives and experiences of the populations, an integrated approach using Western and Indigenous perspectives of health and well-being was used. 

Incorporating Indigenous knowledge within the analysis allowed us to use a holistic, inclusive, and respectful approach and examine impacts at the physical, spiritual, mental, emotional and community levels [37,47]. The extracted results were then used to assist with the development of analytical themes, which were used to assess the impacts of traditional land-based activities on self-reported health. 

The initial data screening and extraction were performed by two of the authors (F.A., and A.M.Z.), with conflicts being reviewed and discussed with L.J.S.T. until a consensus was reached with respect to meeting exclusion and inclusion criteria, and the scope of the review. After the data from each study was extracted, initial themes were identified and evaluated by all authors. 

## 3. Results

### 3.1. Study Selection

Nine studies were selected for this review. In the initial database search (Figure 1), a total of 4067 papers were found through Scopus (n = 84), Web of Science (n= 2016) and PubMed (n = 1967). Search results from the three databases were combined and 216 duplicates were removed. A total of 3851 studies were reviewed at the title and abstract level. The review of these studies sought to identify whether these studies fit our criteria; that is, they involved Indigenous populations, land-based physical activity interventions and our selected health outcomes, as detailed previously. There were 174 papers reviewed at the full-text level for eligibility as per the inclusion criteria. The reasons for exclusion are outlined in the PRISMA flow diagram in Figure 1. Eight qualitative studies were reviewed, and one additional paper was identified for inclusion [24]. Overall, nine studies that focused on the impacts of traditional land-based physical activities on the self-reported health and well-being of Indigenous adults were included in this review. These studies were appraised using the JBI checklist for qualitative research and its ten criteria (Table S2) [45]. Upon appraisal, most of the studies fulfilled each criterion. Of the nine papers, three did not explicitly include a statement addressing the positionality of the researchers [15,22,25]. The remaining six papers explicitly mentioned the positionality of the authors in relation to the respective community [9,10,24,25,32,48]. Overall, we concluded that nine included studies were of high quality, according to the JBI checklist.

### 3.2. Study Characteristics

Of the nine studies identified for inclusion in this review (Table 1), the majority of the studies took place within the USA. Within the USA, studies were with Yup’ik people from the Yukon-Kuskokwim Delta region [15,32], Native Hawaiians from an unspecified region in Hawaii [24,25], the Choctaw Tribe from an unspecified region in Oklahoma [10], Navajo from San Juan County, New Mexico [11] and the United Houma Nation, or Houma, from Louisiana. The remaining two studies took place in Canada. 

These studies were with Urban Indigenous (the term “Aboriginal” was used by Iwasaki and Bartlett [22], as it was appropriate in Canada at the time, however the term Indigenous is now used preferentially [49]), which included First Nations and Métis and was in an unspecified region [22] and the *Uqsuqtuurmiut* in *Uqsuqtuuq* (Gjoa Haven, Nunavut) [48]. Types of land-based physical activity discussed included traditional and subsistence practices such as hunting, fishing, and gathering of food and medicine [11,15,32,48]. Additionally, a total of four studies discussed various forms of ceremonial practices such as hula [24,25], the walking of sacred trails [9,10], and mixed ceremonial practices, including dance and art [22].

A variety of methods (Table 1) were used in these studies to capture the diverse perspectives and experiences of participants. These methods included interviews [9,10,14,24,25,48], focus groups [9,10,11,22,24,32], questionnaires [22] and workshops [48]. Two of these studies offered focus groups and interviews in the Yup’ik language [15,32]. These results were then translated to English by the study authors [32], or another Yup’ik speaker [15]. A third study had some interviews completed in Inuktitut, with translations by an Elder [48]. Four of the included studies explicitly mentioned using a community-based participatory research approach [9,10,24,25] which incorporates community members during every aspect of the research.

### 3.3. Thematic Analysis

Amongst the nine studies, five overarching descriptive themes were identified (Table 2). Subsequently, nineteen analytical themes identified the impacts and challenges of Indigenous adults engaging in land-based physical activities.

#### 3.3.1. Land-Based Physical Activities 

In all nine studies, promoting engagement in land-based physical activities was an effective intervention strategy when addressing health and well-being concerns raised by communities (Table 2). When questioned about their health and well-being, participants responded that subsistence lifestyles were at the core of their beliefs [32], along with cultural identity [22] and ceremony [9,10,25,32]. Subsistence activities were prevalent in five of the papers [9,11,15,32,48]. These activities included hunting, fishing, berry picking, growing and harvesting plants and medicine papers [9,11,15,32,48]. These activities defined the roles and identities of individuals and were an important aspect of life within communities. When asked about components of Yup’ik wellness, one participant exclaimed how *“Berry picking, gathering plants, cutting and drying fish constitute an important role for women and are a key component of Yup’ik/Cup’ik*” ([15], p. 45). Gardening was an activity that helped to address the availability of food and offered many other social and health benefits [11]. Understanding the core values of communities and individuals provides a more in-depth understanding of potential positive impacts or barriers to these land-based physical activities. In one Yup’ik community, when asked about what was central to the lifestyle of conceptions of wellness, one participant responded, *“To live a good life? Cultural activities, the lifestyle itself”* ([32], p. 351). Similarly, another participant responded, *“Besides doing our subsistence work?”*([32], p. 353). These activities were seen as synonymous to health as one participant explained, *“We know that hunting and fishing is one the best means of being healthy, and going out on the land and what not, they tell us that that helps your mind”* ([48], p. 552). This emphasizes the importance of traditional lifestyle and how high of a priority it is for the health and well-being of individuals. The ubiquitous mention of subsistence activities within dialogues being ‘the way of life’ or the ‘lifestyle’ demonstrates the level to which it has been embedded in the lifestyle and the traditional roles within the communities. 

Another form of land-based physical activity observed in four of the studies was traditional ceremonial practices [9,10,22,24,25]. Practices such as hula [24,25], ceremonial walks [9,10], pow-wows and other forms of dance [22], were seen to incorporate all aspects of health and well-being. Hula is an Indigenous dance of Hawai’i, which is taught by *Kumu hula* and is comprised of controlled rhythmic movements which allude to the meaning or poetry of accompanying songs and chants [24,25]. As stated by one *Kumu hula*, *“I see a really great relationship between hula and health in all aspects of health, not just physical health, but mental and emotional health, spiritual health...automatically, you are addressing all, all aspects of the human being and the human being’s health”* ([25], p. 22). This supports the widespread Indigenous teaching that health is a holistic concept and, illustrates the ability of ceremonial practices to affect each aspect of health. When participating in a ceremonial walk *Yappalli,* to pray respects to their ancestors, one participant stated, *“By walking [the Trail] I recognized what my ancestors had put before me…It’s not that I just belong but now I have a responsibility, and now that I’ve walked it I have an accountability”* ([10], p. 12). This demonstrates the deeper connection and feelings of purpose that participants gain in their lives when partaking in ceremonies. A participant partaking in another Houma ceremonial walk, spoke about their connection with their ancestors, *“I think in spaces like we had… nature provided us with everything we needed, and I think they would have wanted us to know how to use that, not just the animals or fishing, but like the plants, the remedies, the just being at peace in nature. I think that’s what they would have wanted”* ([9], p. 10). Retracing their ancestors’ footsteps through these trails allowed them to draw strength and illustrates the idea of balance between the culture and land. Other practices such as pow-wows and other forms of dance [22], were described as moving participants away from their comfort zones to address some of the changes and barriers they face to health [10]. Partaking in these subsistence and ceremonial activities transcend Western behavioral approaches to health and connect to the experiential practices associated with traditional Indigenous health knowledge [10]. Overall, subsistence and ceremonial activities were shown to be central to the traditional roles and lifestyles of individuals, however, participants also highlighted deep-rooted and systemic reasons for a lack of participation.

Participants not only discussed community, but individual-specific challenges they have faced which have led to decreased participation, with the most apparent barriers stemming from colonization and settler colonialism [22,48]. Colonization and it’s systemic effects have been directly linked to the cumulative effects on Indigenous peoples [22]. Evidence of deep-rooted racism in schools, law enforcement, employment practices, and health programs have created many barriers and thus impacting their overall stress and well-being [22,32]. As one participant expressed, *“I think a lot of people would prefer to go back and utilize more traditional food. The state and federal government restrict us from hunting the traditional food that we’ve always ate”* ([32], p. 357). Similarly, *Uqsuqtuurmiut* practices of caribou hunting are regulated through quotas [48]. As explained by one participant, *“The law that’s been imposed on the Inuit people in terms of hunting, the law itself is not connected to the Inuit culture, the Inuit way of living... the law applied to Inuit people is not feasible...because it’s working against their culture and the way of life”* ([48], p. 556). These examples highlight the role government regulations on land, water and wildlife play on creating barriers for subsistence activities [32,48]. The second most common challenge and barrier that was discussed was the lack of knowledge transfer in communities [11]. Regarding gardening, lack of knowledge transfer was seen as a huge barrier where statements like *“we used to jar a lot of fruits and vegetables and I was small then I see them do it …I never learned how to do it” illustrated the loss of traditional knowledge between generations”* ([11], p. 227). This diverse scope of knowledge, which is characteristic of the communities, was previously passed down through oral stories, traditions, and experienced-based learning. These activities were also a way to spend time with family, Elders and other community members outside, as opposed to indoors with nonphysical activities like watching television [11]. The increased reliance of technology was frequently mentioned as a factor leading to the decreased transfer of Indigenous knowledge. As one participant expressed, *“The older Eskimo people, they had to earn their food. It wasn’t mailed in on the plane and they had to use different transportation, so they were healthier... they walked out to go hunting. They didn’t have a snow machine to jump on”* ([32], p. 357). This overall increased reliance on technology has led to a level of inactivity and was shift from subsistence (e.g., hunting) to a wage economy (e.g., sitting behind a desk) [32]. In regions such as *Uqsuqtuuq* (Gjoa Mirja Hirvensalo Haven, Nunavut), the changes brought about by colonization has led to the belief of land-based activities being stressful and difficult. As stated by one participant, *“Our Elders would love to see us encouraging more Inuktitut and more hunting and fishing but, like everybody lives in a settlement now so they’d rather live in town than go out there because it’s an easier life*” ([48], p. 552–553). The sedentary lifestyles and socio-economic challenges that come with subsistence lifestyles continue to influence these beliefs and cause a decrease in participation. Overall, these challenges have led to an increased prevalence of disease and poorer health, which has also become a barrier due to limited physical capacity for participating in ceremonies and subsistence activities [10,24]. Although these challenges were identified, there was a consistent recognition of the benefits on physical, mental, spiritual and emotional health and community engagement amongst all nine studies.

#### 3.3.2. Physical Health

Physical health was a descriptive theme common in all the studies (Table 2). Participation in subsistence land-based activities and traditional ceremonial practices provided an opportunity for increased physical activity in communities. Traditional ceremonial practices such as hula [24,25] and the walking of ceremonial trails [9,10], were forms of traditional activities which were a source of physical activity. The integration of hula into cardiovascular health interventions was effective as it allowed for greater physical conditioning through *“Breathing exercises, spine alignment, foot placement, and stretching”* ([25], p. 22). Participants increased their level of physical activity by partaking in hula under the guidance of *Kumu hula* teachers [24,25]. As one *Kumu hula* explained, *“Now, if we look at hula for its physical benefit, I don’t think any hula dancer will say it doesn’t benefit them physically. It gets them more in tune with their bodies”* ([25], p. 22). Therefore, partaking in hula helps with physical conditioning of an individual through its various movements and training. Doing this under the guidance of an experienced *Kumu hula* is also reiterated as an important factor as it ensures that participants are maintaining correct form and gradually building strength [24,25]. Another type of ceremonial practice was the walking of historic and culturally significant trails. This activity encouraged and motivated behavioral changes amongst individuals by allowing them to go past the Western approaches to health and well-being and learn the multi-faceted approach to health through traditional Indigenous knowledge [10]. As stated by one participant, *“We know we need to take our vitamins. We know we need to go for a walk, but that intervention needs to resonate with our heart and our body. The walk is one way to get to that body place”* ([10], p. 11). Other ancestral ceremonies such as stomp dance were also viewed as important for a recommitment to one’s health. As one participant expressed, *“I think it only strengthened my identity of being Houma”* ([9] p. 11). Engagement not only furthered cultural continuity, but also initiated further interest in other ceremonial practices such as plants and medicines used for healing and ancestral ceremonies such as stomp dance [9]. Overall, participants encountered situations that encouraged them to think of their health beyond physical conditioning and focus on their emotional and mental health, along with a greater emphasis on nutrition in their daily lives [9,10].

Decreased physical activity and poor nutrition were identified as modifiable risk factors for diabetes [22,32]. One reason for these changes was the over-reliance on technology [32]. As described by one participant, *“I never went out by boat, four wheeler, nor by snow machine…I walk all the time remembering what my mother used to tell me: people that go out walking in the wilderness: sickness doesn’t get to them as much...Now I quit going for long walks… I stay home most of the time”* ([32], p. 356). These technological advances, as well as the increasing prevalence of store-bought food were cited as factors for decreased physical activity. As stated by another participant, *“The older Eskimo people, they had to earn their food. It wasn’t mailed in on the plane and they had to use different transportation, so they were healthier”* ([32], p. 357). This reliance on technological advances and store-bought food has created a shift from consumption of traditional food. When discussing factors which contributed to health and health-aging, participants discussed how activities such as berry picking and cutting fish provided them with a modified range of physical activity as it required them to use their muscles while lifting and gathering [15]. Gardening, whether community gardens or in their residence, provided an opportunity for more strenuous activity and decreased reliance on store-bought foods [11]. As one participant recalled, *“When we had a garden every summer, how we did not have a lot of store-bought snacks but we would go to the garden to snack on what was growing”* ([11], p. 227). Similarly, when discussing the caribou hunting traditions of previous generations, one participant stated, *“Like our parents, our grandparents, all they did every day was to try and see where they can find the meat… it was a work and work thing year-round… The old people always said that your body has to continually move all the time; it has to move, move, in order to have your body healthy.”* ([48], p.552). Another participant who expressed how the harvesting of food provided a different type of activity. He stated *“When I was out there the caribou that they caught, every time they skinned it, cut it up. It wasn’t tiring to me, because it was something different from my daily job working”* ([48], p. 555). Partaking in activities such as hunting and fishing also provided access to more healthy, nutritious food for many individuals who rely on store-bought foods [11,15,32]. A major concern amongst community members was for younger generations who prefer market foods and don’t know how to obtain subsistence foods [15]. One Elder shared that growing up poor they had to obtain their own food, and were rarely sick, *“People that go out walking in the wilderness: sickness doesn’t get to them as much”* ([32], p. 356). Participants described the quality of traditional foods as tasting better, being more nutritious, and keeping hunters full for longer periods [32]. Participants also shared stories of moose meat lasting longer than meats from the store, with subsistence foods such as sourdock (a perennial herb) and dried fish, being more affordable and providing more energy and nutrition during long winter hunts [32]. Overall, there was a consensus amongst individuals that the traditional processes of obtaining food provided a high-level of physical activity and provided access to nutritious food, overall contributing to well-being [15,22,32].

#### 3.3.3. Spiritual Health

Spiritual health was a descriptive theme common in five of the studies [10,22,24,25,32]. Spiritual health encompasses all aspects of health including physical, mental, and emotional health, therefore, from a *Kumu hula* perspective, is one of the most important [24,25]. Spiritual health is not widely recognized in a Western context as an aspect of health; however, for many Indigenous populations, it is a key element of health and well-being as it allows for the expression of culture and life [10,24,25,32]. As one female participant described, *“Spirituality connects us with our personal identity, our history, our cultural, our connections with the land, our family, our relations and behaving that everything has a spirit”* ([32], p. 355). These complex connections, which are formed through spirituality, depict values which are foundational to the well-being of the Indigenous adults in the studies. Within ceremonies such as hula, understanding the meanings of songs, poetry, and chants as an expression of life, culture and health is emphasized to connect the individual through reciprocity with themselves and their surrounding environment and community [24,25]. With the practice of Hula, the word Aloha was commonly recognized as being *“Instrumental in the relationship of hula to health, connecting the Kumu hula to students and the students to each other”* ([25], p. 23). This demonstrates the interconnected relationship of the impacts experienced through engagement in traditional ceremony by Indigenous adults. The connections formed through engagement in spiritual practices were also consistent with the theme of reciprocity, or mutual dependence [9,10,24,25]. Participants prayed for guidance to help them through their difficulties and feeling the need to reciprocate by taking care of themselves [10]. These impacts, beyond the increase in physical activity, are observed at a community and familial level. One participant expressed, *“I have a responsibility to the tribe, so it’s not only just being a member, but I have a responsibility towards our preservation and that other people have those experiences and making those connections*” ([9], p. 12). These connections, apparent through participation in ceremonial practices, provided participants with a sense of support, guidance and purpose [9,10,24]. Challenges were cited as motivation and an opportunity to make behavioral changes in one’s life [10,24]. As stated by one female participant, *“All those things, all those unknowns- trying to find a place of comfort… I had to find a way to, to make it okay within myself and not knowing [how, because] there were so many variables”* ([10], pp. 7–8). Taking part in a ceremonial walk and being in an unknown place, physical discomfort and a sense of unease led her to seek ways to make sense of her experience. Difficulties and hardships as an offering were specified as an explicit Indigenous response which set apart culturally relevant interventions from other types of wilderness education or therapy [10]. One participant stated, *“And if I [approach] this as a ceremony I have a certain set of expectations and I just know things are going to be hard… And so you don’t shy away from it; you just accept it and you figure out how you can learn from it”* ([10], p. 10). Opportunities to overcome these discomforts and the unknowns allowed participants to move beyond self-doubt and other personal narratives they may have had about themselves and their abilities [10]. In a similar study, partaking in the Houma ceremonial walk was seen as rediscovering the connection of the land to historical, ancestral place. As one participant stated *“think our ancestors envisioned for us to be able to be proud of who we are, of course… I think they would have been saying, look, learn and continue to pass down. Otherwise, their journey would have been in vain. Their struggles would have been vain. Their lives would have been in vain, if we don’t pass it on down”* ([9], p. 10). Partaking in the ceremonial walk of their ancestors provided them with a space to reflect upon their Houma culture and connect to their relations. Ceremonial practices which took place during their trip including traditional dance, basket weaving and group prayers were also seen as an opportunity to reconnect *“to one another and the earth at a spiritual level*” ([9], p. 11). Overall, spiritual health was regarded as a benefit that connected individuals to their personal identity, history, culture, land, family, and overall brought about a sense of rejuvenation and renewal [22,32].

#### 3.3.4. Emotional and Mental Health

Emotional and mental health was a descriptive theme common to all the studies (Table 2). Acculturative stress from colonization impacted access to subsistence food through government restrictions; this was a barrier in many communities [11,15,48]. Western lifestyles have significantly changed the way of life of some Indigenous communities, increasing feelings of stress and disconnect [32]. As expressed by one participant when being on the land, “*you lose a sense of well-being in the community...but when you go out on the land, you feel a sense of freedom and the worries you had...in the community seem to disappear”* ([48], p. 553). Similarly described by another participant, *“[I]n the springtime when it’s warmer out, when [we] go out camping and stay out of town, it makes [us] feel lighter in [our] minds”* ([48], p. 553). Being on the land created spaces for self-reflection and connection to one’s ancestors and self. One participant stated, *”Not necessarily because we were there as a group, but like for myself in like just quiet times and just feeling very rooted and very connected”* ([9], p. 10). The sense of freedom physically and emotionally is alluded to along with feelings of joy and relaxation. Besides health-related stressors from living with diseases such as diabetes, stress experienced by participants was also a part of a broader structural life context of socio-economic, historical, and political [22]. When speaking about government restrictions, one participant exclaimed, *“For our life that is pretty much like in between a rock and hard place. This kind of situation causes a lot of stress for some people. Stress can affect your health, too, mentally and physically”* ([32], p. 257) This barrier refers to the inability to practice one’s traditional culture, as well as that of the dominant group and results in limited access to subsistence resources and opportunities for wage earning employment. Activities such as visiting camp provided a sense of rejuvenation, relaxation and renewal for participants who otherwise expressed experiencing feelings of stress and anxiety from illnesses such as diabetes being a barrier to wellness [22]. Participants expressed the detrimental effects of diabetes as one participant stated, *“Having to watch what you eat. This being on a diet, watching your sugars— that’s real stress. I had to give up a lot of stuff I like to eat. There’s not being able to do the things I used to be able to do”* ([22], p. 329). The stress of management and prevention led participants to feelings of denial, helplessness, and stigma about the illness [22]. 

Culturally appropriate leisure such as Indigenous art, pow-wows and other forms of dance also provided them with satisfaction and stress relief as stated by an individual, *“It takes away stress. We dance together, and that was a lot of fun”* ([22], p. 331). Overall, increased engagement in cultural and communal activities brought about many positive benefits. As one participant exclaimed, *“Belonging to groups, just for get-togethers. They’re all my culture, they’re all Native. Even just going sitting there, listening to them talk… I always come home with such a light feeling because they share so many wonderful stories with me, and it makes me feel good and happy”* ([22], p. 331). During ceremonial walks, one participant noted their feelings of happiness from being in nature “*Just kind of laughing, giggling and just to kind of be there just with the nature right there”* ([9], p. 10). This illustrates the positive emotions associated with group activities with those sharing the same culture. These approaches towards well-being gave importance to the experiences of participants and allowed them to engage within cultural spaces physically, mentally, emotionally, and spiritually [9,10,24]. 

Another common theme present in the reflections of participants was the holistic approaches providing methods of coping with stress and improved health. Increasing focus and connection during ceremonial practices such as a hula is emphasized through cognition, such as increasing memory retention [25]. The personal development through practice and education from partaking in ceremonies is also highly emphasized [48]. As stated by one participant, *“[E] xperience makes a person have the feeling of being independent and that they are able to do something in a correct manner”* ([48], p. 554). Managing stress and encouraging positive thoughts and emotions were common amongst participants across studies [10,24,25]. In ceremonies such as Hula, *“unconditional love (aloha) is established and expected between teacher and student and amongst students”* ([24], p. 107). The bonds created, and the commitment to the ceremony are a means of management and reduction of stress and negative emotions [24,25]. The recognition of mental health benefits observed during and after activities was also seen as motivation [10]. For some participants, the physical activities led them to challenge themselves mentally, as the experiences caused them to break down. As one participant expressed, *“I had to be forced to face whatever the deepest thing that was going on at the moment was… I was forced to deal with thoughts like that. Feelings of not being worthy”* ([10], p. 8). Though these were negative emotions, the participant went on to explain that these emotions forced her to deal with issues that she may not have dealt with in a medical or therapeutic setting [10]. During *Yappalli*, the walking of the ceremonial trail, one participant stated, *“Before [balance] was a very abstract concept for me. Something I was aiming for but didn’t actually think was a possible goal to achieve. And on the trip I actually felt like there were definite moments when I felt like my body and my mind were in balance”* ([10], p. 11). The recognition of this balance that was achieved, and the types of experiences were a successful motivator for behavioral health changes [10,24,25].

#### 3.3.5. Community Engagement 

Community engagement was a descriptive theme common in all studies (Table 2). Community bonding created an opportunity for the development of social supports through engagement in group subsistence and ceremonial practices. Collective activities such as hunting, bring about feelings of unity and harmony amongst community members. As illustrated by one participant, *“Because of a lack of food at times, everybody would gather to one area and share food. And after the meal they would tell stories as well as play any games... one of our Inuit games... The reason why I mentioned that is because having a gathering of that type creates a harmony between people to live a good life as well as a happy life and to be one with each other”* ([48], p. 555). The interconnectedness with the land, community and oneself encompasses the various attributes of health and well-being. Within ceremonies, such as hula, there was an emphasis by *Kumu hula* on unconditionally supporting each other and creating a comfortable and relaxed atmosphere [25]. As stated by one *Kumu hula “when they come in, it has to feel like they’re coming into a home”* ([25], p. 23). Dancing was a way to connect students with *Kumu hula* and each other to create more social supports and bonds [24,25]. These bonds that were created provided support for participants, which helped with increasing participation and engagement [24,25]. The connection that was developed between the dancers had an effect on their overall dancing and reinforced their bond. As one *Kumu hula* expressed, *“I personally like to see that kind of thread working throughout the rows [of dancers], that’s when you know that they are clicking. And they’re all together in that kind of mental state, which strengthens that bond”* ([25], p. 23). Ceremonies such as the walking trails also strengthened the connections amongst participants, as some stating that it *“felt like family”* ([9], p.10). Many of the narratives in these studies reiterate a sense of vitality from reconnecting with one’s social and natural environment and having community-based activities. Statements such as *“We need to have more community activities. I mean that involve everybody, not just a certain age group. Just do it all together…have something that everybody can do”* ([32], p. 358), stressed the importance of inclusivity within the ceremonies. 

Another benefit of engagement, which was very important, was cultural preservation and upholding valued traditions. For some, the ceremonial practices such as walking of the trail introduced hardships which led *“to new respect for the strength of ancestors and the opportunity to honor that strength through healthy living”* ([10], p. 12). Similarly, a participant took part in the Houma ceremonial walk stated, *“I felt proud to be related to somebody or a group of people who have survived so much and are still so strong”* ([9], p. 10). This not only provided them with a sense of appreciation and respect for their ancestors and history, but also a sense of responsibility for cultural continuity. While incorporating traditional activities into interventions, there was great emphasis on the involvement of community members to ensure that practices remained culturally appropriate [22,25]. As one *Kumu hula* stated, *“If the whole program can be designed appropriately for the health benefits as well as the hula benefits, then I think it is a wonderful way to bring people back into their culture if they don’t already have a strong connection”* ([25], p. 24). Working together in group and community settings was also beneficial in the establishment of community gardens and allowed for more access to nutritious food for the community and increased opportunity for communication and knowledge transfer [11]. Participants expressed, *“The way things are going right now the changes in society and what not on the reservation, might not be a bad idea to have a community project, that way it would increase communication”* ([11], p. 227). Subsistence activities were a way to foster communication and increase engagement within the community, with their culture and with Elders.

In many settings, Elders provided intergenerational knowledge transfer [11,15] and were a crucial aspect of the community [15,22,24,25,32]. When discussing community gardens, respondents had stated their respect for Elders and knowledge transfer as a way of learning new techniques [11]. Recognizing health issues faced by the community raised awareness of the loss of intergenerational traditional knowledge transfer, and the importance of connection to Elders within communities [11]. When reflecting on the experiences of participants, spending time with Elders brought about more awareness to concerns about cultural change, which was impacting traditions, and how to maintain and preserve them for future generations [10,15,22,32]. When learning and partaking in hula, strong bonds were created between class participants and *Kumu hula* because of the supportive environment it created and the valuable transferrable lessons which were taught of discipline, forgiveness, and conflict resolution [24,25]. Additionally, concerns were raised in some communities of the drastic change in lifestyle and a decline of traditional practices and knowledge transfer, causing health to deteriorate [11]. When speaking about Elders, one participant stated, *“They taught them how they should live their lives in the future, to live a good life so that they would live long…they would pick up moss and wash themselves saying that they want to live a good, long life… It was like wiping away the future sickness”* ([15], p. 47). Overall, Elders were seen as an important resource in communities for maintaining cultural identity through language and knowledge transfer [15,32]. Additionally, like the supports established through with *Kumu hula* through hula [24,25], engagement in subsistence activities such as hunting fostered support systems with Elders. As stated by one participant, *“like way back when, they never really used to do that [suicide], and today it’s kinda hard. There’s lots of teenagers already done it before… And I think to get more help, to start off with Elders... they have such strong words when they love you so much, like they talk to you”* ([48], pp. 554–555). Ultimately, there were similarities amongst all studies regarding the importance of engagement in traditional activities at a community and individual level, and an overall desire for increased participation. Although each paper sought to address different challenges and health disparities, there was a consensus regarding the array of benefits of engaging communities to incorporate traditional activities into health promotion strategies. As expressed by Elders and participants, involving community members throughout all stages of health and well-being interventions allowed for them to remain culturally appropriate and respectful to the traditions [9,10,22,24,25]. Overall, using various forms of traditional activities in these studies provided an opportunity for participants to be more engaged and empowered using culturally relevant methods to address their health and well-being.

## 4. Discussion

This systematic review addresses knowledge gaps by identifying the potential benefits of partaking in traditional land-based activities for Indigenous adults. Looking through the lens of the two-eyed seeing approach *(Etuaptumumk)* [36], our review identified key impacts of traditional subsistence and ceremonial practices on the self-reported health of Indigenous adults. Using a thematic analysis, impacts on community engagement, spiritual, physical, and emotional and mental health were observed. Examining the perspectives of Indigenous adults identified barriers and challenges which have led to a decrease in participation [9,11,15,32,48]. These barriers, stemming from colonization, included increased government regulations [11,32,48], the decreased transfer of Indigenous knowledge [11,15,32,48] and an overall increased reliance on technology [11,15,32]. The inclusion of practices relating back to ancestral and familial traditions when addressing health disparities, was an important factor in influencing the participation and overall outcomes for Indigenous adults [9,10,11,48]. Indigenous adults participating in subsistence and ceremonial activities shared their accounts of the array of positive benefits they experienced. These benefits included developing deeper connections to their traditions [9,32,48], increased levels of physical activity [10,24,25,48], improved nutrition [11,15,32] and developing increased community connections and social supports [9,22,24,25,32,48]. Identifying the benefits and barriers, allowed for a deeper understanding of the health disparities faced by individuals and communities. These findings show an array of benefits when traditional Indigenous knowledge is incorporated with Western approaches to health to address some health disparities faced by Indigenous communities. 

The findings from this study are consistent with the increasing awareness of the notion that health and well-being are more than a biomedical concept but are also determined by social circumstances and cultural contexts [37,47]. The use of traditional Indigenous knowledge has reinforced cultural identity as a core value in many Indigenous communities [9,10,11,15,29,31,32,50]. Historically, Indigenous knowledge has been transmitted through storytelling, art, and by visual and experience-based teachings [24,31,32,50,51]. Within our review, this knowledge system, passed within and between generations, created a foundation for the well-being of the communities [10,11]. A systematic review by Teufel-Shone, et al. [52] looking at the impact of physical activity interventions in USA and Canada identified the lack of programs designed from an Indigenous perspective using culturally relevant health promotion and scientifically proven methods. The review also found that many times, these programs are also not practical or acceptable for the community to implement [52]. This shortcoming can create difficulty when addressing health issues as the physical component may be addressed through current Western science-based approaches, however, components of spirituality, emotional and mental health which may cause decreased physical activity and poorer nutrition are not addressed. The combination of these approaches stresses the importance of using integrative approaches [41]. Interventions which use the two-eyed seeing approach present an opportunity to preserve cultural values and traditions [2,31,32,52], increase community relations and social supports, provide more opportunities for Elder engagement [6,10,11,29,50], and opportunities for empowerment of individuals [10]. Similarly, in a study by Bazzarre [53] which aimed to adapt physical activity interventions to address obesity in culturally diverse populations, found that it is important to identify factors which promote healthy changes within populations to establish best practices within communities. For example, a study by Proust, et al. [54] highlighted the importance of traditional food consumption amongst First Nations populations with respect to combatting chronic diseases. Given the documented health inequities amongst Indigenous communities, it is crucial to take more effective and sustainable approaches towards addressing these health disparities. In our review, adults described positive experiences of being on the land, such as deeper connections with the land and ancestral traditions and using this, to influence their own health behaviours. Adults participating in ceremony and subsistence activities identified their challenges and effective ways to modify their own behaviours, to obtain more long-term benefits and sustainable changes to their behaviours [11,15,32]. To promote and maintain these changes, establishing community support networks for individuals has also been shown to be a beneficial factor for these health interventions [39,50]. Additionally, involving key community members and the use of Indigenous knowledge provided a more positive, sustainable, and effective method for addressing health disparities within Indigenous populations. Overall, interventions must seek to decolonize knowledge systems by recovering and renewing traditional, non-commodified cultural patterns such as mentoring and sustaining intergenerational relationships [55].

When working with Indigenous communities which have historically faced impacts of oppression, racism, and colonization, it is important to have methodologies such as focus groups and interventions, which provide them with opportunities to share their perspectives and experiences [12,13,15,19,24,31]. Similarly, a study by Pelletier, et al. [56], concluded that institutional, political, and cultural barriers must be considered when developing interventions for Indigenous communities. Our review identified impacts of colonization [9,11,15,32,48], increased use of technology [9,10,11,15,32,48], increase in availability of store-bought food [10,11,15], and decreased participation from youth [9,11,25] were all factors identified which contributed to decreased physical activity and poorer nutrition within communities. Previous studies also identified that programs which were developed by staff who lacked advanced training or materials, were not adapted to meet the cultural needs of the community and were ineffective [41]. Therefore, consistent with the findings of other studies [6,21,30] there is a need for involvement of community members throughout all stages of the intervention to allow for these barriers to be more effectively addressed at the government and institutional levels. Furthermore, as stated by Bazzarre [53], current physical activity interventions are designed for communities and not adapted to ‘real-world’ practices despite having significant impacts. Therefore, it is important that such programs are designed keeping in mind the resources available to the communities to effectively implement and sustain the interventions. Involving community members and community expertise during all stages of development, implementation and evaluation is crucial to provide effective and sustainable interventions. Future studies should employ a cost-benefit analysis with a sustainability plan, and feedback from community members to reap the optimal benefits.

Another important factor which must be taken into account when establishing culturally relevant on-the-land programs is the importance of traditional ecological knowledge (TEK). The term TEK was introduced into research in the 1980s by academics to raise awareness of the value of Indigenous knowledge which had been accrued over generations [57,58,59]. The term “traditional” used for this knowledge system describes the cultural continuity which is transmitted through values, benefits and practices of the community [60]; however, it should be emphasized that traditional does not mean static, TEK is dynamic and constantly evolving. Indigenous communities are often seen as knowledge holders of TEK because of their experience and connections with their land [48]. This knowledge from Indigenous knowledge holders provides tremendous insight into areas of wildlife biology, health, and disease ecology [61], to name a few disciplines. Maintaining cultural practices and knowledge is also necessary for sustainable and equitable use of community resources. Practices such as hunting, trapping, and fishing not only provide physical activity and access to nutritious food but help further community and familial connections. Participation in these practices also facilitates the intra- and inter-generational dissemination of knowledge which is crucial for caring for and maintaining the environment [6,7,48]. Thus, the land and connections with the land are of utmost importance to Indigenous people. For example, in the USA, resistance to the Dakota Access Pipeline from the Lakota people was motivated by many factors, one of which being the Lakota cultural and spiritual practices related to the environment. Water in Lakota life is seen as a lifegiving force, referred to as “the first medicine” [62]. Therefore, in many studies examining Indigenous health, impacts on the environment are described as being synonymous to impacts on Indigenous health [6,9,32,48]. Thus, incorporating Indigenous knowledge when addressing health inequities within communities is important as it incorporates different perspectives and experiences within areas such as health promotion [61].

The findings of this review also identify a need for further exploration of community and culturally specific land-based activities for Indigenous adults. Our findings present many benefits of integrating culturally relevant activities into interventions. However, due to the heterogeneity and limited number of studies, it was difficult to examine the differences between ceremonial or subsistence traditional activities. The differences between the types of activities and the benefits of integrating them into health and well-being interventions is community-specific and therefore more studies employing community-based participatory research (CBPR) methods would be beneficial [56]. Many studies have highlighted the importance of CBPR when working with Indigenous communities [34,38,54,56,63]. One of these studies, by Foulds, Bredin and Warburton [63] examined the health benefits of community-based physical activity interventions for Indigenous Canadians. Their findings suggested that community-based interventions were more successful amongst Indigenous populations, with improvements observed in health measures such as waist circumference and an increase in physical among participants of both genders, and across age groups and training programs [64]. Another study by Lines, Yellowknives Dene First Nation Wellness Division and Jardine [50], found that connection with the land has many positive health impacts on Indigenous youth and their family, the overall community and surrounding environment. Furthermore, it was recommended that work with Indigenous communities incorporate land-based components and employ localized strengths and resources of communities [50,64]. This is also becoming more apparent with the increasing awareness on the importance of traditional food systems, which contribute to many aspects of health and well-being [11,63]. One study which used a community-based program to harvest overabundant geese in subarctic Canada found the program strengthened food security, contributed to environmental sustainability, and strengthened social networks and feelings of wellness for participants while on the land [62]. Working closely with community members allows for more effective, and culturally relevant ways to address the health disparities which impact communities [50,54,65]. Therefore, employing more studies using CBPR would help to identify what types of traditional activities would be best suited for health interventions in communities.

Our study was limited as we were unable to examine the differences between community-specific activities by gender and by individuals living in rural or urban settings. A study by Bingham, et al. [66] looking at the gender differences among First Peoples of Canada, identified that gendered health disparities must be framed by discussions of the distinct historical experiences of Indigenous men and women. These experiences are just some of the factors which lead to our understanding of why gender differences in health disparities exist, however as stated by Jen’nan Ghazal and Bridget [67], there is a need for more multi-level and mixed-methods research to allow for a more comprehensive framework dealing with gender as an organizing principle of life that structures opportunities and resources at the individual and contextual levels. Therefore, more studies employing a diversity of communities employing the use of objective biomedical measures such as steps using a pedometer, blood pressure and blood glucose, along with subjective measures such as individual perceptions and experiences to evaluate the impacts on self-reported health taking gender into account are needed [32]. Another factor that must be considered is whether individuals reside in an urban or rural setting. Only one of our studies [22] was distinctly with an urban Indigenous population and identified that the stress experienced in their lives which contributed to diabetes also stemmed from broader structural systems and dynamics. The study found that culturally based forms of leisure were useful when dealing with culturally bound stressors such as racism. These findings were contrary to a study done by Hossain and Lamb [5], which sought to understand the differences of cultural attachment and mental well-being amongst Canada’s rural and urban Indigenous populations. The study found that those people residing in large urban centers that lacked involvement in traditional cultural activities were not impacted, with respect to their level of psychological wellbeing, potentially due to assimilation into non-Indigenous cultures [5]. The findings of both these studies suggested that further research must be done to understand the differences in experiences of urban and rural Indigenous adults, as community settings can drastically change the kinds of barriers faced by rural and urban Indigenous participants in future research.

### Limitations

As determined by the JBI criteria [45], the studies included in our review were of high quality; however, limitations still exist for this review. Our search strategy was developed to be inclusive of the diversity, but the nine studies identified were all situated within the USA and Canada. It is possible that studies were missed due to variations in the terminology used by the communities to identify themselves. Our review also did not provide a definition for land-based physical activities to be as inclusive as possible of the diversity of traditional land-based activities amongst Indigenous communities. Due to the diversity identified amongst the studies, we can acknowledge that we were correct in doing so. Many of the studies did not provide the specific questions asked in their data collection, therefore themes may have emerged depending on the questions that were asked by the researchers. The majority of our studies employed either a community-based participatory research approach [9,10,24,25] or had authors which were members of the respective communities [9,10,11,32]. These methods allow for the engagement of community members to leverage expertise of Indigenous knowledge holders to address community concerns [56] and ensure a balance between research and action benefiting the community and science [67,68]. It is important to note that the small number of studies obtained for this review are reflective of individual beliefs, community-specific health risks and concepts of physical activity and can only be generalized with caution. Furthermore, for this reason it is important to engage community members and those with expertise to allow for increased cultural sensitivity and ensuring that the values and traditions of the communities are maintained [24,25]. Despite the limitations discussed, using integrative methods such as the two-eyed seeing approach ensures Indigenous knowledge, values, and perspectives, of their respective communities, are strongly reflected in the research.

## 5. Conclusions

This systematic review identified the various interrelated impacts of land-based physical activities on the self-reported health and well-being of Indigenous adults. The review analyzed the diverse perspectives and experiences of Indigenous adults using thematic analysis and viewing of Indigenous and Western approaches to health and wellness as complementary. Our analysis identified impacts on physical activity, spirituality, mental and emotional health, along with community engagement. Historical and systemic impacts of colonization were identified as some of the major barriers for the participation in traditional subsistence and ceremonial practices. However, incorporating them into Western approach-based health interventions provided opportunities for increased physical activity, awareness of modifiable risk factors, development of effective stress management techniques, and opportunities for knowledge transfer and social support within communities. As there is great diversity amongst Indigenous populations, these barriers and benefits can only be deemed applicable at a community level. Therefore, understanding the perceptions and opinions of target groups along with the involvement of community expertise is important when developing and implementing interventions. Furthermore, there is a need for more studies incorporating traditional land-based physical activities relevant to other geographic areas, using complementary approaches. Future studies are needed to develop more sustainable, effective, and culturally relevant traditional land-based physical activity interventions that improve self-reported health and well-being in Indigenous populations.

## Figures and Tables

**Table 1:** A Summary of Study Characteristics.

Study	Population; Region	Total Sample Size (N); Sex; Age	Objective	Traditional Physical Activity	Data Recruitment	Methodology
Wolsko, Lardon, Hopkins and Ruppert [32]	Yup’ik; Yukon- Kuskokwin Delta, USA	N = 64 Females and Males Ages ≥18 years	Examining Indigenous conceptions of wellness	Subsistence activities Hunting, fishing, traditional medicine and trapping	Advertisements Word of mouth	Focus groups English and central Yup’ik Language with translations by study authors Authors as moderators and cofacilitation with a community member Nominal group technique for discussion Grounded theory using open and axial coding strategies
Hopkins, Kwachka, Lardon and Mohatt [15]	Yup’ik; Yukon- Kuskokwin Delta, USA	N = 15 Females only Ages 38 to 89	Exploring cultural beliefs about health and aging	Subsistence activities Hunting, fishing, and berry picking	Advertisements (village traditional councils and local health clinics) Word of mouth	Interviews with probes Conducted in English and Yup’ik with translations by a Yup’ik speaker Ethnographic qualitative data gathering methodology
Look, Kaholokula, Carvhalo, Seto and de Silva [24]	Native Hawaiians; Hawaii, USA	N = 23 Focus groups with Females (n = 6) and Males (n = 11) Ages 38 to 72 Interviews with Females (n = 5) and Males (n = 1) Ages 50 to 80s	Integrating multiple community perspectives to inform a design of a hula-based cardiac rehabilitation intervention	Ceremonial Practices Hula	Local community coordinators for interviews Focus group respondents were patients hospitalized for a cardiac event within the past year	Focus groups with coronary artery disease patients Interviews with *Kumu hula* Community-based participatory research Thematic analysis approach
Look, Maskarenic, De Silva, Seto, Mau and Kaholokula [25]	Native Hawaiians; Hawaii, USA	N = 6 Females (n = 5) and Males (n = 1) Ages 50+	Evaluating the impact of hula as part of a clinical intervention including cardiovascular disease prevention and management programs	Ceremonial practices Hula	Through acquaintances or pre-existing relations	Semi-structured interviews Community-based participatory research Thematic analysis
Lombard, Beresford, Ornelas, Topaha, Becenti, Thomas and Vela [11]	Navajo; San Juan County, New Mexico, USA	N = 31 Females and Males Ages ≥18 years	Determining Navajo attitudes related to gardening	Subsistence lifestyle practices Gathering and harvesting	Advertising (fliers) Word of mouth	Focus groups Conducted until saturation was achieved and no new themes emerged Thematic analysis
Schultz, Walters, Beltran, Stroud and Johnson-Jennings [10]	Choctaw Tribe; Oklahoma, USA	N = 6 Females Ages 21 to 49	Applying principles of wilderness experience programming and Indigenous knowledge in an exploratory intervention designed to address health disparities in a tribal community	Ceremonial practices Walking the trail of tears (*Yappalli*)	Purposive sampling	In-depth interviews pre and post walk. Focus groups during the walk Participants travelled across Arkansas over nine days over various physical environments Community-based participatory research Thematic analysis Historical trauma framework
Johnson-Jennings, Billiot and Walters [9]	The United Houma Nation; Louisiana, USA	N = 20 Females Ages 18 to 45	Identify a United Houma Nation health framework, by co-developing a community land-based healing approach in order to inform future community-based health prevention program	Ceremonial Practices Walking to retrace the forced migration of UHN ancestors (*Hina*) Subsistence lifestyle practices Hunting, fishing and gathering	Local community researcher Community citizens across six parishes Social media sites The community newsletter	Daily activities integrated into the route Pre and post interviews Two focus groups Body composition analysis pre and post walk Community-based participatory research
Iwasaki and Bartlett [22]	Urban Indigenous (First Nations & Métis); Region not specified, Canada	N = 26 First Nations females (n = 8) and males (n = 9) Métis females (n = 9) Ages 26 to 69	Gain insight into lived experiences of urban Indigenous Canadians with diabetes in stress and coping through leisure	Ceremonial practices Dance, ceremonies, and visiting reserves and camps	Purposive sampling Advertising (Posters displayed at an Indigenous health center)	Focus groups Questionnaire for socio-demographic background information Use of professional moderator (Female, non-Indigenous) Resilience Framework Phenomenology analysis
Robertson and Ljubicic [48]	*Uqsuqtuurmiut; Uqsuqtuuq* (Gjoa Haven, Nunavut), Canada	N = 39 Females (n = 12) and males (n = 27) Most contributors Elders	Addressed community priorities: caribou health, caribou food (vegetation) quality and access, changing lifestyles, cultural values and skills, Inuit health and diet, and Elder and youth land camps	Subsistence lifestyle practices Hunting and fishing	Contributors recommended by planning committee Selected based on experience	Three summer land camps Interviews Inuktitut translations done by Elder Result-verification workshops to discuss interpretations

**Table 2:** A Summary of the Thematic Analysis.

Descriptive Themes	Analytical Themes
Land-based Physical Activities	Subsistence activities [9,11,15,32,48] Central to the lifestyle and defined roles [9,11,15,32,48] Ceremonial practices [9,10,22,24,25] Barriers stemming from colonization and settler colonialism [9,11,22,32,48] Reliance of technology [11,32,48]
Physical health	Subsistence lifestyle better for health and wellbeing [9,10,11,15,32,48] Influenced behavioral changes [9,22,24,25,32] Lack of inactivity due to over reliance on technology and store-bought foods [11,15,32]
Spiritual health	Expression of culture and life [9,22,24,25] Challenges faced used as motivation for behavioral changes [10,24] Reciprocity [9,10,24,25,32]
Emotional and Mental health	Psychological impacts of diabetes and other health outcomes seen as a barrier to wellness [22] Stress due to impacts of colonization or acculturative stress [9,11,15,32,48] Holistic approach provided of methods of coping with stress and encouraged reflects of health [9,10,22,24,25] Mental health benefits were motivation [10,24,25]
Community Engagement	Importance of social supports and community bonding [11,22,24,25,32,48] Maintaining cultural identity [9,10,15,22,25,48] Importance of Elders for knowledge transfer [9,11,15,22,24,25,48] Concerns of rapid cultural change and loss of valued traditions [15,32]

## Supplementary Materials

The following are available online at https://www.mdpi.com/article/10.3390/ijerph18137099/s1, Text S1. An adapted version of the United Nations system to identify Indigenous peoples [43], Table S1. Search Strategy, Table S2. Quality assessment adapted from the JBI critical appraisal checklist [45].
