# Supplementary material for: The Impact of Land-Based Physical Activity Interventions on Self-Reported Health and Well-Being of Indigenous Adults: A Systematic Review
Source: Int J Environ Res Public Health. 2021 Jul 2;18(13):7099. doi: 10.3390/ijerph18137099 (PMC8296996; doi:10.3390/ijerph18137099)
Supplement: Supplementary file 1 [file ijerph-18-07099-s001.zip › ijerph-1199681-supplementary materials.pdf]

International Journal of
Environmental Research and Public Health

MDPI

Supplementary Material:

Text S1. An adapted version of the United Nations system to identify Indigenous peoples [43]

The following system has been developed by the United Nations to provide an understanding of the term Indigenous:

1. Self- identification as Indigenous peoples at the individual level and accepted by the community as their member
2. Historical continuity with pre-colonial and/or pre-settler societies
3. Strong link to territories and surrounding natural resources
4. Distinct social, economic, or political systems
5. Distinct language, culture, and beliefs
6. Form non-dominant groups of society
7. Resolve to maintain and reproduce their ancestral environments and systems as distinctive peoples and communities

Table S1. Search Strategy. The following table includes detailed search strategies for the three databases searched.

Database	PubMed	Scopus	Web of Science
Totals	*1967*	*84*	*2016*
Search Strategy	(((“Indigenous People*” OR “Aboriginal*” OR “Trib*” OR “Native” OR “First Nation*” OR “Inuits” OR “Metis” OR “Métis” OR “Oceanic Ancestry Group” OR “American Native Continental Ancestry Group”) AND (“Program*” OR “Intervention” OR “Activ*” OR “Land*” OR “Land-based” OR “bush” OR “water*” OR “subsistence” OR “traditional*” OR “hunt*” OR “fish*” OR “gather*” OR “forag*” OR “Life style”)) AND ((“well being” OR “well-being” OR “wellness” OR “Self Perception*” OR “Health perception” OR “Diagnostic self evaluation”) OR (“Hypertension” OR “Heart Rate” OR “Blood Pressure” OR “Diabetes Mellitus, Type 2” OR “Hyperglycemia” OR “Insulin Resistance” OR “Cortisol” OR “Stress” OR “Sleep”)))		You searched for: (ALL=(((“Indigenous People*” OR “Aboriginal*” OR “Trib*” OR “Native” OR “First Nation*” OR “Inuits” OR “Metis” OR “Métis” OR “Oceanic Ancestry Group” OR “American Native Continental Ancestry Group”) AND (“Program*” OR “Intervention” OR “Activ*” OR “Land*” OR “Land-based” OR “bush” OR “water*” OR “subsistence” OR “traditional*” OR “hunt*” OR “fish*” OR “gather*” OR “forag*” OR “Life style”)) AND ((“well being” OR “well-being” OR “wellness” OR “Self Perception*” OR “Health perception” OR “Diagnostic self evaluation”) OR (“Hypertension” OR “Heart Rate” OR “Blood Pressure” OR “Diabetes Mellitus, Type 2” OR “Hyperglycemia” OR “Insulin Resistance” OR “Cortisol” OR “Stress” OR “Sleep”)))) AND LANGUAGE: (English)<br>Refined by: [excluding] PUBLICATION YEARS: (2020) AND DOCUMENT TYPES: (ARTICLE OR BOOK CHAPTER) AND RESEARCH AREAS: (ENVIRONMENTAL SCIENCES ECOLOGY OR SOCIOLOGY OR ANTHROPOLOGY OR HEALTH CARE SCIENCES SERVICES OR AGRICULTURE OR SOCIAL SCIENCES OTHER TOPICS OR PSYCHOLOGY OR BEHAVIORAL SCIENCES OR ETHNIC STUDIES) AND WEB OF SCIENCE INDEX: (WOS.SCI OR WOS.SSCI) AND DOCUMENT TYPES: (ARTICLE OR BOOK CHAPTER) AND WEB OF SCIENCE CATEGORIES: (ENVIRONMENTAL STUDIES OR ENVIRONMENTAL SCIENCES OR SOCIAL SCIENCES BIOMEDICAL OR PUBLIC ENVIRONMENTAL OCCUPATIONAL HEALTH OR HEMATOLOGY OR MULTIDISCIPLINARY SCIENCES OR IMMUNOLOGY OR ENDOCRINOLOGY METABOLISM OR PSYCHIATRY OR CARDIAC CARDIOVASCULAR SYSTEMS OR AGRICULTURE MULTIDISCIPLINARY OR SOCIAL SCIENCES INTERDISCIPLINARY OR

			BIOCHEMICAL RESEARCH METHODS OR ANTHROPOLOGY OR PHYSIOLOGY OR PSYCHOLOGY MULTIDISCIPLINARY OR HORTICULTURE OR PERIPHERAL VASCULAR DISEASE OR BEHAVIORAL SCIENCES OR FOOD SCIENCE TECHNOLOGY OR EDUCATION EDUCATIONAL RESEARCH OR BIODIVERSITY CONSERVATION OR SUBSTANCE ABUSE OR BIOLOGY OR LINGUISTICS OR SOCIOLOGY OR GERIATRICS GERONTOLOGY OR NUTRITION DIETETICS OR INTEGRATIVE COMPLEMENTARY MEDICINE)<br>Timespan: All years. Indexes: SCI-EXPANDED, SSCI, A&HCI, CPCI-S, CPCI-SSH, BKCI-S, BKCI-SSH, ESCI.
Language filter	English	English	English
Publication Dates	Until 16 June 2021	Until 2021	Until 2021
Other Filters Used	Species specified as Human	n/a	Web of science core collection citation indexes: Science Citation Index Expanded (SCI-EXPANDED) --1900-present & Social Sciences Citation Index (SSCI) --1900-present<br>Document type: Refine for only Article and Book Chapter<br>Research areas: Environmental Sciences Ecology, Sociology, Anthropology, Agriculture, Social Sciences Other Topics, Psychology, Behavioural Sciences or Ethnic Studies<br>Web of science categories: Environmental Studies, Environmental Sciences, Social Sciences Biomedical, Public Environmental Occupational Health, Hematology, Multidisciplinary Sciences, Immunology, Endocrinology Metabolism, Psychiatry, Cardiac Cardiovascular Systems, Agriculture Multidisciplinary, Social Sciences Interdisciplinary, Biochemical Research Methods, Anthropology, Physiology, Psychology Multidisciplinary , Horticulture, Peripheral Vascular Disease, Behavioural Sciences, Food Science Technology, Education Educational Research, Biodiversity Conservation, Substance Abuse, Biology, Linguistics, Sociology, Geriatrics Gerontology, Nutrition Dietetics

Table S2. Quality assessment adapted from the Joanna Briggs Institute (JBI) critical appraisal checklist [45].

Appraisal Checklist Criteria	Wolsko, Lardon, Hopkins and Ruppert [32]	Iwasaki and Bartlett [22]	Lombard, Beresford, Ornelas, Topaha, Becenti, Thomas and Vela [11]	Schultz, Walters, Beltran, Stroud and Johnson-Jennings [10]	Hopkins, Kwachka, Lardon and Mohatt [15]	Look, Maskarenic, De Silva, Seto, Mau and Kaholokula [25]	Look, Kaholokula, Carvhalo, Seto and de Silva [24]	Johnson-Jennings, et al. [9]	Robertson and Ljubicic [48]
1. Is there congruity between the stated philosophical perspective and the research methodology?	Yes	Yes	Yes	Yes	Yes	Yes	Yes	Yes	Yes
2. Is there congruity between the research methodology and the research question or objectives?	Yes	Yes	Yes	Yes	Yes	Yes	Yes	Yes	Yes
3. Is there congruity between the research methodology and the methods used to collect data?	Yes	Yes	Yes	Yes	Yes	Yes	Yes	Yes	Yes
4. Is there congruity between the research methodology and the representation and analysis of data?	Yes	Yes	Yes	Yes	Yes	Yes	Yes	Yes	Yes
5. Is there congruity between the research methodology and the interpretation of results?	Yes	Yes	Yes	Yes	Yes	Yes	Yes	Yes	Yes
6. Is there a statement locating the researcher culturally or theoretically?	Yes	Unclear	Yes	Yes	Unclear	Unclear	Yes	Yes	Yes
7. Is the influence of the researcher on the research, and vice- versa, addressed?	Yes	Yes	Yes	Yes	Yes	Yes	Yes	Yes	Yes
8. Are participants, and their voices, adequately represented?	Yes	Yes	Yes	Yes	Yes	Yes	Yes	Yes	Yes
9. Is the research ethical according to current criteria or, for recent studies, and is there evidence of ethical approval by an appropriate body?	Unclear (REB not mentioned	Unclear (REB not mentioned	Yes	Yes	Yes	Yes	Yes	Yes	Yes

10. Do the conclusions drawn in the research report flow from the analysis, or interpretation, of the data?	Yes	Yes	Yes	Yes	Yes	Yes	Yes	Yes	Yes
